# Resection of anterolateral midbrain cavernous malformation via orbitozygomatic transsylvian pretemporal approach with uncal resection

**DOI:** 10.3171/2019.7.FocusVid.19153

**Published:** 2019-07-01

**Authors:** Krishna C. Joshi, Hamid Borghei-Razavi, Varun R. Kshettry

**Affiliations:** Department of Neurosurgery, Cleveland Clinic, Cleveland, Ohio

**Keywords:** cavernoma, brainstem, fiber tract, oculmotor-tentorial window, carotid oculomotor, video

## Abstract

Brainstem cavernomas are benign, angiographically occult, low-flow lesions and constitute 18%–35% of intracranial cavernomas.^4,6^ They are known to have an annual rupture risk of 2%–6%,^2,5^ and once symptomatic, they frequently cause progressive neurological morbidity. A 22-year-old lady presented with progressive profound neurologic deficits from three distinct hemorrhages over 2 months. Surgery was indicated given the aggressive natural history, and the lesion now presented to the surface with displacement of corticospinal tracts noted on diffusion tensor imaging.^1,7^ We describe a surgical technique via an orbitozygomatic transsylvian pretemporal approach with uncal resection to open the oculomotor-tentorial window and resect the lesion.^3^

The video can be found here: https://youtu.be/j5yYp4OsaRc.

## Transcript

**FIG. 1.**
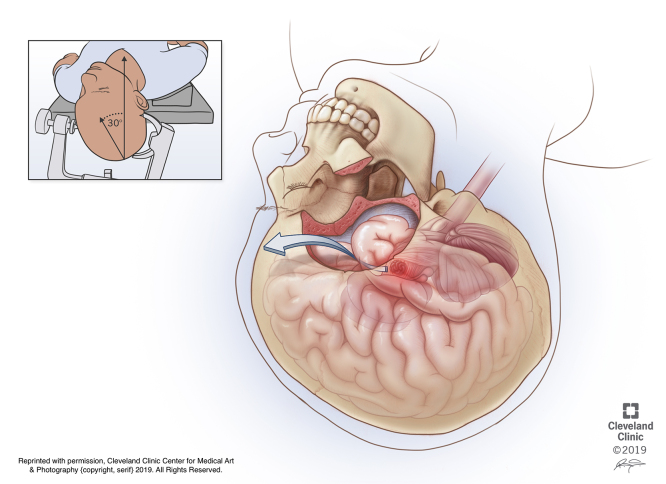
Right orbitozygomatic craniotomy has been performed. The transsylvian surgical trajectory to the anterolateral midbrain is highlighted by the arrow. Top left inset: Patient positioning. Reprinted with permission, Cleveland Clinic Center for Medical Art & Photography. © 2019. All Rights Reserved.

We will be reviewing a case of an anterolateral midbrain cavernous malformation resected via an orbitozygomatic transsylvian pretemporal approach with uncal resection. Twenty-two-year-old right-handed lady presented with headache, mild lower facial weakness, and mild left hemiparesis (4+/5). CT and subsequent MRI demonstrated a hemorrhagic lesion in the right cerebral peduncle most consistent with a cavernous malformation. The lesion did not appear to come to the pial surface first, and considering the deficits were mild and this was her first presentation, we elected for observation. Ten days later she re-presented with double vision, new mild right cranial nerve palsy, worsening in her left-sided hemiparesis, and sensory abnormalities. CT demonstrated mild increase in size of hemorrhage. One month later she presented with progressive worsening, increased sleepiness, mild cranial nerve III palsy, left facial droop. Her motor movement decreased to 3 proximal and 0 distally in her upper limbs. She also had diminished light touch and was unable to walk. MRI demonstrated expansion of the hemorrhagic lesion with new extension into the thalamus. The lesion now clearly came to the surface. We elected to proceed to surgery, in view of aggressive clinical course and deterioration and now that the lesion was coming to the pial surface. DTI was performed to track the corticospinal tracts **(2:05)**, and in the upper right inset you can see that the corticospinal tracts have been displaced laterally, which opened up a trajectory in the anterolateral midbrain. The patient was positioned supine with the malar eminence as the most prominent point. Next, a standard frontotemporal incision was made, and we performed a two-piece standard orbitozygomatic craniotomy using standard cuts (Fig. 1). We believe the OZ would afford us the ability to look up at the most superior aspect of the cerebral peduncle. After removing the reminder of sphenoid wing, we are cutting the orbitomeningeal band and we will begin by opening the sylvian fissure. Fine-tipped bipolars can be used to spread apart bands of arachnoid while watching for any inadvertent stretch of vessels **(2:49)**. In addition to cutting the arachnoid bands, the microscissors can be similarly used to spread the arachnoid, and with the tips closed the end can be used as a sharp dissector and the shaft as a retractor **(3:00)**. One of the keys to this approach is the wide sylvian fissure exposure to maximize exposure of frontal and temporal lobes. A cotton ball can be used to keep frontal and temporal lobes separated **(3:24)**. Our dissection is carried proximally to the origin of supraclinoid carotid artery. We have identified the ICA terminus and the M1 and are performing wide release of the M1 branch **(3:45)**. We then want to untether the frontal lobe from the anterior cranial fossa **(3:56)** and are dissecting all the arachnoid along the optic nerve **(4:00)**. This is the arachnoid of the opticocarotid cistern **(4:10)**. We will also dissect the arachnoid in the interoptic space to allow frontal lobe release. The contralateral optic nerve is visualized; we identified the A1 and optic tract and are releasing tethers to the frontal lobe **(4:26)**.

One of the important steps in the temporopolar approach is the wide release of the anterior temporal artery from its arachnoidal attachments. This will allow maximal displacement of the temporal lobe posteriorly and laterally without compromise of the anterior temporal branch **(4:41)**. From the dura of the anterior clinoid, the anterior petroclinoidal ligament can be followed posteriorly to identify the oculomotor nerve **(4:57)**. We will then follow the oculomotor to its origin from the brainstem. Here we can see the ICA, PComm, and the P2a segments, and the third nerve just ventral and the midbrain dorsal **(5:04)**. For purpose of orientation, here is a view from superficial to target, which is the oculomotor-tentorial window; the uncus blocks further access to this surgical space **(5:08)**. Here the sylvian vein entry into the sphenoparietal sinus is identified **(5:24)**. When retracting the sylvian vein posteriorly, care must be taken to avoid injuring the sylvian vein drainage or the anterior temporal artery. To open up the oculomotor-tentorial window further, the uncus can be partially resected **(5:53)**. After partial resection of the uncus, the arachnoidal pia of the uncus are cut and the uncus is mobilized posteriorly. We are now able to fully expose the anterolateral surface of the midbrain, and we find an area of abnormal color medially and normal-appearing tissue laterally **(6:12)**. Papavarine was placed on proximal vessels prophylactically **(6:22)**. Cortisectomy was made on the discolored area of the brainstem, and further dissection was performed until the lesion was encountered **(6:30)**. In this case, much of the hematoma was organized and firm and was removed piecemeal **(6:45)**. Within the crural cistern, the upper limit of dissection is the anterior choroidal artery and we exposed, and we ended our cortisectomy up until the upper limit to fully access the lesion **(6:56)**. Superficially in the cavity we encountered more hematoma and we removed this piecemeal. Deeper and medially in the cavity we encountered what we felt was the cavernoma proper **(7:20)**. Although this was adherent, we spent time dissecting along its margins. The areas of the cavity were felt to be more consistent with organized hematoma, and much of this was removed as safely possible. Normal borders were encountered, last remaining bits of tumor were removed, and final hemostasis was achieved **(8:23)**. The wound was closed in the standard fashion. The frontal sinus lateral wall was repaired with a pericranial flap. She was extubated immediately postop and initially had an unchanged neurological exam. Her alertness and strength started improving over next 48 hours and began to walk within 1–2 weeks. By 8 weeks, had complete resolution of all neurological deficits and was able to run on a treadmill and return to work. MRI at 3 months shows satisfactory postop changes **(8:55)** and she remains well.
